# Supplementary material for: Assembly and Cellular Exit of Coronaviruses: Hijacking an Unconventional Secretory Pathway from the Pre-Golgi Intermediate Compartment via the Golgi Ribbon to the Extracellular Space
Source: Cells. 2021 Feb 26;10(3):503. doi: 10.3390/cells10030503 (PMC7996754; doi:10.3390/cells10030503)
Supplement: Supplementary file 1 [file cells-10-00503-s001.pdf]

Cell biology glossary

BFA; Brefeldin A— an antiviral compound produced by the fungus *Penicillium brefeldianum*. Rapidly removes membrane-bound COPI coats and is therefore commonly used in cell biological experiments to disassemble the cisternal Golgi stacks.

COPI; Coatomer protein I—a multisubunit protein complex that operates in vesicle formation at IC and Golgi membranes. A well-known function of the COPI vesicles is in retrograde transport; that is, in recycling of proteins and lipids to the ER. COPI coats are also important for Golgi integrity.

ER; Endoplasmic reticulum—an extensive membrane network, where proteins and lipids destined for other organelles of the endomembrane system, the cell surface, or secretion are synthesized, folded and modified, and thus prepared for transport along the secretory pathway.

ERES; ER exit site—a specialized subdomain of the ER where newly synthesized proteins and lipids are packed into transport vesicles to enter the secretory pathway.

ERGIC; ER-Golgi intermediate compartment—an alternative acronym for the IC (see below).

ERGIC-53/p58—the first identified marker of the IC/ERGIC. A lectin-like cargo-receptor that operates in the export of selected glycoproteins from the ER and protein recycling at the ER-Golgi interface.

Golgi ribbon—the typical mode of Golgi organization in vertebrate cells, where multiple cisternal Golgi stacks in the perinuclear region are laterally connected by so-called noncompact zones.

IC; Pre-Golgi intermediate compartment—the first post-ER compartment of the secretory pathway, which besides traditional ER-Golgi communication has been shown to operate e.g., in protein folding, Golgi-independent trafficking and autophagy.

Rab GTPases—key regulators of membrane traffic, which specifically associate with different organelles of the endomembrane system, and are thought to define their identity.

RE; Recycling endosomes—a widespread a membrane network operating in membrane recycling from early and late endosomes to the cell surface. In many cell types REs accumulate in the pericentrosomal region, forming the endocytic recycling compartment (ERC).

VTC; Vesicular tubular cluster—a morphological term frequently used for the IC/ERGIC.
